# Downregulation of stathmin 1 in human gallbladder carcinoma inhibits tumor growth *in vitro* and *in vivo*

**DOI:** 10.1038/srep28833

**Published:** 2016-06-28

**Authors:** Jiwen Wang, Yanli Yao, Yue Ming, Sheng Shen, Nan Wu, Jiaqi Liu, Han Liu, Tao Suo, Hongtao Pan, Dexiang Zhang, Kan Ding, Houbao Liu

**Affiliations:** 1 Department of General Surgery, Zhongshan Hospital, General Surgery Institute, Fudan University, Shanghai 200030, China; 2 Glycobiology and Glycochemistry Lab, Shanghai Institute of Materia Medica, Chinese Academy of Sciences, Shanghai 201203, China; 3 PET-CT Center, Cancer Hospital, Chinese Academy of Medical Sciences, Peking Union Medical College, Beijing 100021, China; 4 Beijing Key Laboratory for Genetic Research of Skeletal Deformity, Beijing, China; 5 Department of Orthopaedic Surgery, Peking Union Medical College Hospital, Peking Union Medical College; Chinese Academy of Medical Sciences, Beijing 100730, China; 6 Department of Breast Surgical Oncology, Cancer Hospital of Chinese Academy of Medical Sciences, Beijing, 100021, P. R. China; 7 Department of General Surgery, The Fifth People’s Hospital of Shanghai, Fudan University, Shanghai 200240, China

## Abstract

Gallbladder carcinoma (GBC) is a highly lethal malignancy of the gastrointestinal tract. Despite extensive research, the underlying molecular mechanism of GBC remains largely unclear. Stathmin 1 (STMN1) is an important cytosolic protein associated with microtubule stability that was reported to be involved in tumorigenesis. Up to our knowledge, its role in gallbladder carcinoma has not been analyzed. In this study, we found that STMN1 was significantly highly expressed in GBC by immunohistochemistry (IHC). Further research demonstrated that silencing of STMN1 inhibited cell growth *in vitro*. Moreover, knockdown of STMN1 induced apoptosis and delayed G2/M phase transformation in GBC cells. Our data support a rationale for further studies that the silencing of STMN1 may regulate the activity of p38 MAPK kinase and p53/p21 signal pathway. Besides, xenografted gallbladder carcinoma cells growth were significantly impaired after STMN1 was silenced *in vivo*. These results suggested that STMN1 played an important role in cell proliferation and migration. This provided a potential clue for investigating the therapeutic target in GBC.

GBC is the most common malignancy of the biliary and gastrointestinal tracts^1^. The incidence and distribution of GBC differ across worldwide. For example, the prevalence of GBC in South and East Asia is considerably higher than that in Europe and America^2^. To date, surgical resection is the main treatment option for GBC. However, most patients are not candidates for curative resection at the time of diagnosis. Moreover, there is a high risk of recurrence of GBC after surgery^3^. Despite the advances in chemotherapy, the treatment for GBC remains challenging due to its poor prognosis. The mean survival of patients with GBC is reported to range between 5.2 and 24.4 months^4^,^5^. Although recent studies have reported that accumulated progress of GBC treatment, the exact molecular mechanism underlying this progression is still unclear^6^. Besides, the potential therapeutic target was needed for the precision treatment for GBC patients^7^.

STMN1, an 18-kDa cytosolic protein, which is also known as p17, p18, p19, 19 K, oncoprotein 18, prosolin, and metablastin, is an important regulator of microtubule stability. It exists broadly in cytoplasm and plays an important function in regulating cell proliferation, differentiation, motility, clonogenicity and survival^8^. STMN1 was reported to be overexpressed in various cancers, such as leukemia^9^, and breast^10^, ovarian^11^, gastric^12^, lung^13^, and prostate cancers^14^. Elevated expression of STMN1 in cancer is invariably associated with the proliferation and matastasis of cells^15^. As a microtubule (MT)-destabilizing protein, the silencing of STMN1 caused a delayed G2 phase in several cancer cell lines^16^,^17^. Alli *et al*.^18^ found that silencing of STMN1 in human breast cancer cells induced the overexpression of cleaved caspase-9 and cleaved caspase-3. Besides, the silencing of STMN1 enhanced the chemotherapy sensitivity of glioma cell^19^, gastric cancer cell^20^, and HEL JAK2^V617F^ cell lines^21^ by inducing apoptosis. These phenomena prompted us that STMN1 may provided negative impact on the effect of chemotherapy. Nevertheless, the expression of STMN1 in GBC has not yet been elucidated, and the underlying molecular mechanism is still unknown.

In this study, we reported the role of STMN1 in GBC. The results showed that downregulation of STMN1 greatly influenced the progression of GBC, and STMN1 might be a potential therapeutic target for GBC.

## Results

### STMN1 was highly overexpressed in GBC tumor samples

**Figure 1:**
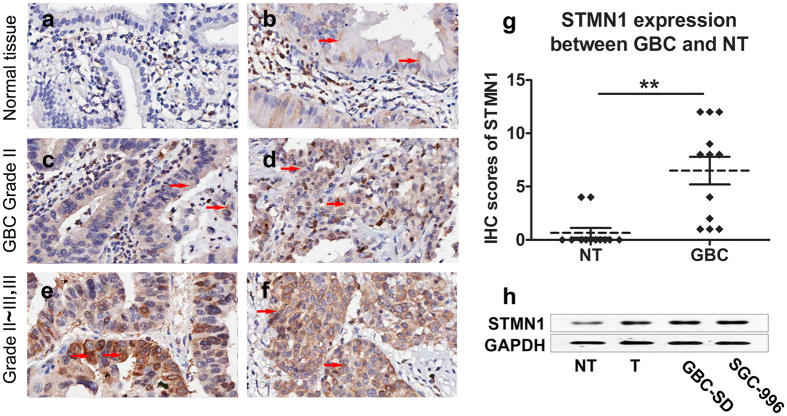
The expression of STMN1 was increased in GBC. Representative images of STMN1 immunostaining in adjacent normal tissues (**a,b**) and different stages of GBC tissues (GBC tissue of Grade II (**c,d**); GBC tissue of Grade II~III, III (**e,f**)). Twelve pairs of GBC and pericarcinomatous tissues were sectioned and subjected to immunohistochemistry, as described in Methods section (Origianl magnification is 200×). Red arrows indicated STMN1 positive signal. The expression levels of STMN1 between GBC and adjacent tissue were shown in the scatter plot (*p* = 0.0003, significance was determined using Student’s *t*-test) (**g**). The STMN1 expression level was scored by semi-quantitative scoring system as described in Methods section. The protein level of STMN1 was detected in two GBC cell lines (GBC-SD, and SGC-996) (**h**), with GBC and pericarcinomatous tissue were used as positive and negative controls, respectively. GAPDH was used as a control for protein loading. NT: adjacent normal tissues; T: tumor tissue. GBC: Gallbladder carcinoma.

The expression of STMN1 in GBC has not yet been reported. Therefore, we first analyzed twelve pairs of tumor and pericarcinomatous tissues by IHC. The characteristics of the patients were listed in Supplementary Table S1. STMN1 expression level of the tissues was scored by a semi-quantitative scoring system, as described in Methods section. The results showed that all of the twelve pericarcinomatous tissues were negative (IHC score <5) for STMN1 expression (Fig. 1a,b). While the expression of STMN1 in five of the twelve GBC tissues were negative and the other seven GBC tissues were positive (IHC score ≥5) for STMN1 expression (Fig. 1c–f). STMN1 expression of GBC tissues was significantly higher than the expression of pericarcinomatous tissues (*p* = 0.0003, Fig. 1g). High expression level of STMN1 was significantly associated with high pathological grade (*p* = 0.018). However, similar finding was not present with TNM stage in the 37 cases (*p* = 0.338, Supplementary Table S2).

### Silencing of STMN1 inhibited GBC cells growth

**Figure 2:**
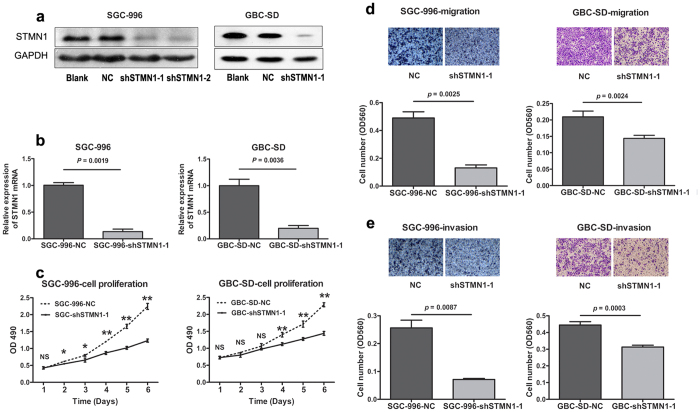
ShRNA of STMN1 suppression inhibited the growth, migration and invasion of SGC-996 and GBC-SD cells. SGC-996 and GBC-SD cells were transfected with vector control (NC) or shSTMN1-1. 72 h later, the protein of each cell line was extracted. The expression of STMN1 in the two cell lines was detected by WB (**a**) and qRT-PCR (**b**), respectively. Then, shSTMN1-1 was selected randomly for further research. The growth curve was tested by MTS assay (**c**). Migration (**d**) and invasion (**e**) assays of SGC-996 and GBC-SD cells were performed 24 h after incubated. Results were shown as means ± SEM from three independent experiments. NS: Non-significant; NC: Negative control. *p < 0.05; **p < 0.01. Significance was determined using Student’s *t*-test.

Western blot was used to evaluate the background expression level of STMN1 in GBC cell lines. The results showed that elevated expression of STMN1 was observed in two cell lines (SGC-996 and GBC-SD) compared to the pericarcinomatous tissue (Fig. 1h). The two cell lines were chosen as models to explore the functional role of STMN1 in GBC. SGC-996 and GBC-SD cells were then infected with STMN1 mRNA-specific lentiviral shRNA vectors (shSTMN1) and non-silencing GV248 control vectors. STMN1 expression was markedly downregulated at protein (Fig. 2a) and mRNA level (Fig. 2b) in the cell lines infected with shSTMN1. Two shRNA vectors targeting to STMN1 (shSTMN1-1 and shSTMN1-2) were used to silence STMN1 expression in SGC-996 cell. The results of Western blot showed that the expression level of STMN1 in shSTMN1-1 and shSTMN1-2 groups were similar. And shSTMN1-1 was selected randomly for further research.

The frequent upregulation of STMN1 in GBC tissues and cell lines suggested that this gene might play an important role in GBC. To investigate the effects of STMN1 deficiency, MTS assay was conducted and cell growth curves were obtained. The results showed that the viability of shSTMN1-1 reduced significantly compared to that of the control, from day 2 after infection in SGC-996 cells, from day 4 in GBC-SD cells (Fig. 2c).

### Stable knockdown of STMN1 expression inhibited migration and invasion of GBC cells

To determine the effect of STMN1 on cell metastasis, trans-well migration and invasion assays were performed *in vitro*. The results showed that cells transfected with shSTMN1-1 displayed a weaker migration ability compared to that of the negative control group. The lower chamber of the shSTMN1-1 group had significantly less cell than that of the control group. (Figure 2d, *p* = 0.0025 in SGC-996 cells, *p* = 0.0024 in GBC-SD cells). Moreover, the suppression of STMN1 significantly reduced the invasion ability of SGC-996 (*p* = 0.0078) and GBC-SD (*p* = 0.0003) cells through Matrigel test (Fig. 2e). In summary, the results demonstrated that STMN1 played an important role in promoting GBC cell migration and invasion.

### Stable knockdown of STMN1 expression induced apoptosis and G2/M arrest of GBC cells

**Figure 3:**
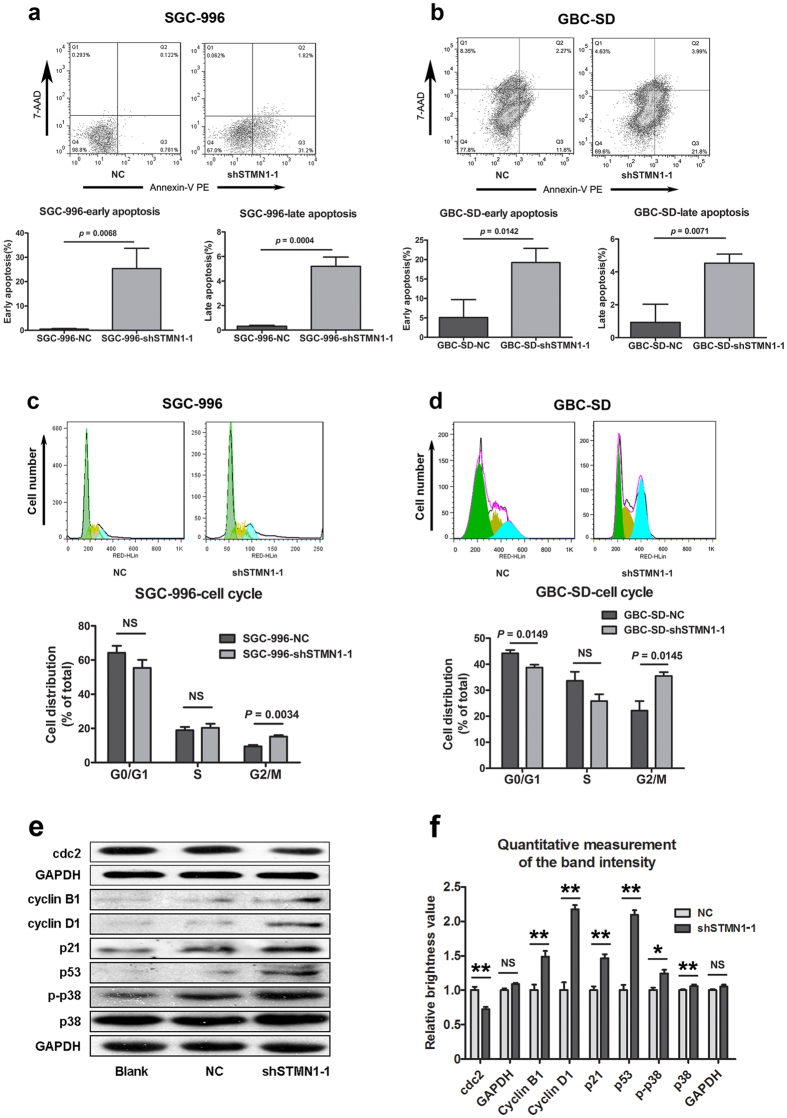
shRNA of STMN1 induced apoptosis and G2/M arrest of SGC-996 and GBC-SD cells. Apoptosis assays were applied after the two cell lines transfected with lentiviral shSTMN1-1 vector or mock-vehicle for 72 h. This assay was determined by Annexin V-PE and 7-AAD staining (**a,b**). Cell cycle analysis after the two cell lines were transfected with lentiviral shSTMN1-1 vector or mock-vehicle for 72 h (**c,d**). Western blot analysis and quantitative measurement of the band intensity after SGC-996 cell was transfected with lentiviral shSTMN1-1 vector or mock-vehicle (**e,f**). The antibodies specific against p38, phosphorylated-p38, p53, p21, cyclin D1, cyclin B1, and cdc2 were used. GAPDH was used as a control for protein loading. Results were shown as means ± SEM from three independent experiments. Significance was determined using Student’s *t*-test.

In order to determine whether the silencing of STMN1 induced apoptosis, AnnexinV-PE/7-AAD kit was used to measure the apoptosis of SGC-996 and GBC-SD cells. As shown in Fig. 3a,b, the early and late apoptosis ratios of the shSTMN1-1 groups were significantly higher than those of the control groups. In addition, the cell cycle analysis indicated that the SGC-996 and GBC-SD cells were arrested at the G2/M phase after STMN1 silencing (Fig. 3c,d, *p* = 0.0034 in SGC-996, *p* = 0.0145 in GBC-SD). Moreover, results of Western blot suggested that the phosphorylation of p38 as well as the expressions of p21 and p53 increased in the shSTMN1-1 group compared to that in the negative control group. The expression of cell cycle protein cdc2 decreased, while that of cyclin B1 and D1 increased after STMN1 knockdown (Fig. 3e). These observations suggested that STMN1 was involved in the cell cycle.

### Stable silencing of STMN1 in SGC-996 cell line decreased tumor growth *in vivo*

**Figure 4:**
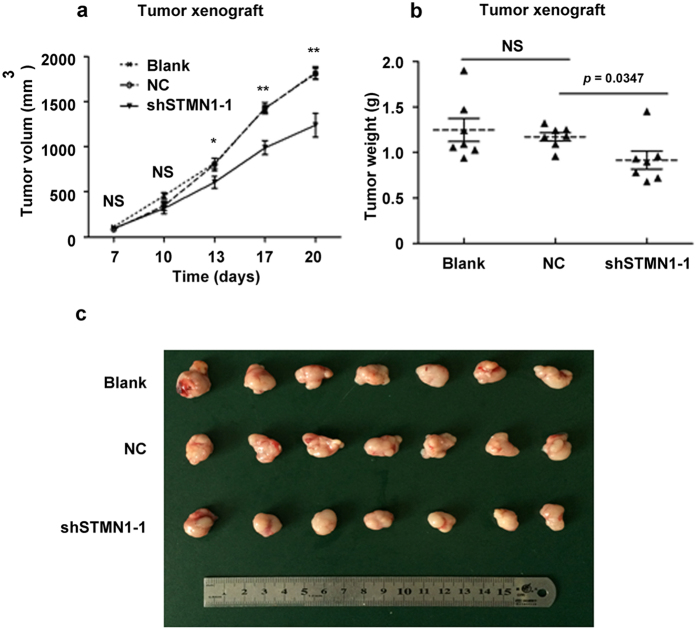
shRNA of STMN1 suppressed the growth of SGC-996 cell xenografts in nude mice. SGC-996 cells from three groups (blank, negative control, and shSTMN1-1) were subcutaneously injected in the armpit of the nude mice, as described in “Methods”. The volumes of the tumors were measured every second or third day. Tumor growth curves were shown (**a**). The mice were sacrificed on the 20^th^ day after injection, and the xenografts were removed and weighed. A statistical plot of tumor weights of the three groups was shown (**b**). Photographs of the xenografts of the each group of mice (n = 7) were shown (**c**). NS: Non-significant; NC: Negative control. *p < 0.05; **p < 0.01. Results were shown as means ± SEM. Significance was determined using Student’s *t*-test.

To further evaluate the effects of STMN1 on the growth of GBC *in vivo*, SGC-996 cells from three groups (blank, negative control, and shSTMN1-1) were subcutaneously injected in the armpit of the nude mice. The tumor volumes of shSTMN1-1 group were signficantly smaller than those of negative control group from day 13 to 20 after the implantation. This result showed that the growth rate of shSTMN1-1 cells was slower (Fig. 4a). After the final measurement on day 20, the mice were sacrificed, and the tumors were excised and weighed. The final tumor weights in the shSTMN1-1 group were significantly decreased compared to the negative control group (Fig. 4b,c, *p* = 0.0347). Taken together, these results suggested that STMN1 was required for tumor growth in a mouse model of GBC.

## Discussion

STMN1 was reported to be overexpressed in various cancers^9^,^10^,^11^,^12^. Elevated expression of STMN1 in cancer is associated with the proliferation and matastasis of a variety of cancer cells^15^. Moreover, the silencing of STMN1 caused a delayed G2 phase^16^,^17^ and an increase of apoptosis rate^18^ in cancer cell lines. Besides, the silencing of STMN1 also enhanced the chemotherapy sensitivity of glioma cell^19^, gastric cancer cell^20^, and HEL JAK2^V617F^ cell lines^21^ by inducing apoptosis. Consequently, these phenomena prompted us that STMN1 promoted the progression of human cancers, and it might be a potential therapeutic target for cancer therapy.

We observed that STMN1 was highly expressed in GBC tissues compared to pericarcinomatous tissues. The results indicated that silencing of STMN1 decreased the growth rate of GBC cells *in vitro* and *in vivo*. Furthermore, the results also suggested that increased apoptosis rate, G2/M arrest, and weaker aggressiveness of GBC cells were detected after silencing STMN1 expression. Therefore, these data implied that STMN1 might be involved in the aggressiveness of GBC, for the reason that it played an important role in cell cycle.

As a microtubule (MT)-destabilizing protein, the silencing of STMN1 caused a delayed G2 phase in several cancer cell lines^16^,^17^. This is consistent with the report by Alli *et al*.^18^ Likewise, our results showed that the knockdown of STMN1 caused a G2/M phase delay in gallbladder carcinoma as well. This phenomenon suggested that silencing of STMN1 affected cell cycles by a MT cytoskeleton dependent mechanism.

Recently, the possible mechanisms that might elucidate the functions of STMN1 in cancer have attracted increasing attention. Wang *et al*. reported that the silencing of STMN1 resulted in decreased expression of Bcl-2 and survivin proteins, and activation of caspase-3^22^. Alli *et al*.^18^ found silence of STMN1 in human breast cancer cells induced the overexpression of cleaved caspase-9 and cleaved caspase-3. Meanwhile, poly (ADP-ribose) polymerase cleavage, measured by the appearance of the 89-kDa cleavage product, was also increased after the silencing of STMN1. Gain-of-function and loss-of-function study revealed that STMN1 increased the amount of cellular acetylated/stable MTs detected by acetylation of α-tubulin in colorectal cancer cell lines^23^. P53 and p21 were reported to block cells at G2 phase by inhibiting Cdc2^24^. Similarly, our data showed that the expressions of p53 and p21 were increased after silencing STMN1. However, the expression of cyclin B1 was increased as well. This was consequence of the inhibition of cyclin B1 ubiquitination and the accumulation of cyclin B1 at M phase of cell cycle, like Lin *et al*. discovered in human colon carcinoma^25^. Therefore, the results indicated that silencing of STMN1 caused G2/M arrest by p53/p21 pathway in several kinds of cancer.

In summary, our research demonstrated that STMN1 was up-regulated in GBC tissues. Suppressing STMN1 expression inhibited cell proliferation, migration, and invasion *in vitro*, and for tumorigenicity *in vivo* of GBC cell lines. Furthermore, silencing of STMN1 caused G2/M arrest and even apoptosis of GBC cell lines. Since the effect of STMN1 was important in GBC, further research about the relationship between STMN1 expression and clinical prognosis will be required to determine whether STMN1 could serve as a prognostic indicator of GBC. Thus, our findings suggested that STMN1 might be a potential therapeutic target for the treatment of GBC.

## Methods

### Patients and specimens

Specimens of GBC were selected randomly from Zhongshan Hospital, Fudan University (Shanghai, China) between 2007 and 2012. Dissected samples were preserved in formalin immediately after surgery and stored at room temperature. Approval from Zhongshan Hospital Ethics Committee was obtained before the research. All experimental protocols were carried out in accordance with the guidelines approved by the Zhongshan Hospital Ethics Committee. Informed consent was obtained from all of the patients in this study.

### IHC analysis

Formalin-fixed GBC tissues were embedded in paraffin, and 4-μm-thick sections were cut and mounted on slides. After deparaffin and antigen recovery, the slides were washed thrice in peroxidase blocking solution (DakoCytomation, Carpinteria, CA, USA). The slides were then incubated with rabbit anti-human STMN1 polyclonal antibodies (1:600; Cell Signaling Technology, MA, USA) overnight at 4 °C, followed by incubation with a secondary antibody (UltraSensitive SP; Fuzhou Maixin Biotech. Co., Fuzhou, China) at 25 °C for 30 min. The immunolabeled slides were visualized by diaminobenzidine for 5 min, counterstained with hematoxylin, and observed under microscope (Olympus CX31; Olympus, Japan).

The IHC results were independently evaluated in a blinded manner by two pathologists. The signal was assessed by a semi-quantitative scoring system, which represented the percentage of positive tumor cells and the intensity of staining. The intensity of staining of cells was scored and graded as follows: ‘0’ (negative), ‘1’ (faint yellow), ‘2’ (yellow or deep yellow), and ‘3’ (tan or brown). The proportion score according to the percentage of positively stained cells was as follows: 0 (0–25%), 1 (26–50%), 2 (51–75%), and 3 (76–100%). The expression of STMN1 was measured by multiplying the staining intensity by the percentage of positively stained cells. The samples with a final score ≥5 were defined as high expression, and those with a final score <5 were defined as low expression.

### Cell culture

Human GBC cell line SGC-996 was provided by the Tumor Cytology Research Unit, Medical College, Tongji University, China. Human GBC cell line GBC-SD was purchased from Type Culture Collection of the Chinese Academy of Sciences, Shanghai, China. Both cell lines were maintained in Dulbecco’s modified Eagle’s medium (DMEM) with 10% fetal bovine serum (FBS), 100 mg/mL streptomycin, and 100 units/mL penicillin. The cells were cultured at 37 °C and 5% CO_2_ in a humidified atmosphere. After every 2–3 days, the cells were sub-cultured in 1 mM EDTA and 0.25% trypsin. The cells were routinely screened and were found to be free of mycoplasma.

### Lentivirus-mediated shRNA knockdown of STMN1 gene expression

shSTMN1 and non-silencing GV248 control vector were purchased from Gene Chem Co. (Shanghai, China). GV248 non-silencing control vector was used as an expression control. GV248 cloning vector contained eGFP and elements that were required for packaging of the expression construct into virions, as well as the puromycin-resistant gene. The target sequences of shSTMN1 were 5′-GAAGAGAAACTGACCCACAAA-3′ (shSTMN1-1) and 5′-CTGGAACGTTTGCGAGAGA-3′ (shSTMN1-2). Lentiviral shRNA was purchased from GeneChem Co. (Shanghai, China). For cell infection, viral supernatants and 8 μg/mL polybrene were added to the culture medium and the medium was incubated for 24 h. Green fluorescence was detected 96 h later, and the expression of STMN1 was verified by Western blot and qRT-PCR. Then, shSTMN1-1 was selected randomly for further research.

### Total RNA extraction and qRT-PCR

GBC cells were harvested one week after transfection. Total RNA was isolated using TRIzol reagent (Invitrogen, Carlsbad, CA, USA), in accordance with the manufacturer’s instructions. RNA concentration and purify was determined by BioPhotometer plus (Eppendorf 6132, Hamburg, Germany). One microgram of total RNA was used for reverse transcription by M-MuLV reverse transcriptase (Fermentas, St. Leon-Rot, Germany). The expression of STMN1 was measured by real-time PCR using ABI 7500 Fast Real-Time PCR System (Applied Biosystems, Foster, CA, USA), and three replications of PCR were performed. The primers and amplification conditions of this study are listed in Supplementary Table S3 (amplification conditions: reverse-transcription reaction; 42 °C, 30 min per cycle. PCR cycling conditions: enzyme activation; 95 °C at 10 s per 40 cycles, and annealing and extension at 60 °C for 32 s).

### Protein extraction and Western blot

The cells were washed with phosphate-buffered saline (PBS; Sangon Biotech, Shanghai, China) and homogenized in RIPA lysis buffer (Beyotime Institute of Biotechnology, Hangzhou, China) on ice for 15 min. The supernatant was obtained after centrifugation at 12,000 × *g* for 30 min. Protein concentration was measured by BCA assay (Beyotime), and the acquired protein samples were stored at −80 °C until use. Equal amounts (20–40 μg) of proteins were mixed with the loading buffer and then electrophoretically separated by SDS-polyacrylamide gels before being transferred onto nitrocellulose filter membranes (Pall Corp, East Hills, NY, USA). In order to block nonspecific binding sites, the membranes were incubated with 5% dry milk or BSA dissolved in TBST (tris-buffered saline containing 0.1% Tween 2.0) at room temperature for 1 h. The membranes were then incubated with an appropriate antibody at 4 °C overnight. The expressions of STMN1 and GAPDH were evaluated by incubating the membranes with horseradish peroxidase (HRP)-conjugated anti-mouse or anti-rabbit secondary antibodies (1:5000; Abmart, Shanghai, China), and immunoreactive bands were developed using the EZ ECL Chemiluminescence Detection Kit for HRP (CoWin Biotech, Beijing, China). All experiments were performed in triplicate. GAPDH was used as an internal control.

The antibodies used in this study were as follows: anti-STMN1 (1:500; Cell Signaling Technology, MA, USA), anti-GAPDH (1:10000; Proteintech Group, Chicago, IL, USA), and anti-p-p38, anti-p38, anti-p53, anti-p21, anti-cdc2, anti-cyclin A2, anti-cyclin B1, and anti-cyclin D1 antibodies (all 1:1000; Cell Signaling Technology, MA, USA).

### Cell proliferation assay

GBC cells were seeded into 96-well plates at a concentration of 3 × 10^4^ cells/well. Every day from day 1 to 6, one plate was subjected to assay by adding 20 μL of MTS solution (CellTiter 96 AQueous One Solution Cell Proliferation Assay) to each well, followed by incubation at 37 °C for 2 h. The absorbance at 490 nm was measured with a microplate reader. The experiment was performed in triplicate.

### Migration/invasion assays

For trans-well migration assay, GBC cells were incubated in serum-free DMEM for 2 h after the transfection for 72 h. The cells resuspended in the serum-free media (100 μL, containing 1 × 10^5^ cells) were seeded into the upper chamber of transwell chambers (Corning Costar, Cambridge, MA, USA). The lower chamber contained 500 μL DMEM with 10% FBS. After seeding, cells were incubated at 37 °C for 24 h before staining the inserts with 0.1% crystal violet (Sigma-Aldrich, St. Louis, MO, USA) and 10% methanol (Sinopharm Chemical Reagent Co., Shanghai, China), and then counted under a microscope for quantification. Subsequently, the chambers were washed with 33% ethylic acid and the OD was measured by a microplate reader. The underside of the transwell chamber was coated with 100 μL of Matrigel matrix (1 : 40) at 37 °C for 2 h.

For invasion assay, lower chambers of plates were coated with 10 mg/mL fibronectin overnight at 4 °C before cell seeding.

### Cell cycle analysis

Briefly, GBC cells were seeded into 6-well cell culture plates with 2 × 10^5^ cells/ well, and incubated at 5% CO_2_ and 37 °C for 24 h. Lentiviral vector was used to infect the cells. Four days later, cells were collected and centrifuged at 1,000 × *g* for 5 min, followed by washing with 1 mL PBS pre-cooled at 4 °C. The cells were then suspended in 250 μL of binding buffer to make a concentration of 1 × 10^6 ^cells/mL. The cell suspension was mixed with 5 μL of Annexin V-PE and 10 μL of 7-AAD. Finally, 1 mL of ice cold 70% ethanol was added to the cells and the solution was maintained at −20 °C. Subsequently, a cell suspension aliquot (containing at least 2 × 10^5^ cells) was centrifuged at 300 × *g* for 5 min, washed once with PBS, and suspended in the fluorescent reagent (Muse^TM^ Cell Dispersal Reagent). After incubation at room temperature for 15 min, 400 μL PBS was added to the cell suspension. The cells were then measured using a flow cytometer (BD FACS Calibur; Becton Dickinson, East Rutherford, NJ, USA). The cells were then fixed with 70% ethanol overnight and stained with propidium iodide before analysis during the cell cycle examination. The pancreatin used in this step was EDTA-free.

### SGC-996 xenograft tumor models in nude mice

Six week old female BALB/c mice were purchased from Shanghai Slac Laboratory Animal Co. LTD. The mice were housed in a temperature-controlled, pathogen-free animal facility with light and dark cycles. 4 × 10^6^ SGC-996 cells were subcutaneously injected in the armpit into each mouse. The mice were sacrificed at the 20^th^ day after transplantation, and the xenografts were removed and weighed before being photographed. The animal studies were approved by the Animal Ethical Committee of Zhongshan Hospital. All the methods were carried out in accordance with the approved guidelines.

### Statistical analysis

*T*-test was used to compare the differences between two groups. *P* < 0.05 was considered statistically significant. The results were expressed as means ± S.E.M. All statistical analysis was performed by using SPSS 18.0.

## Additional Information

**How to cite this article**: Wang, J. *et al*. Downregulation of stathmin 1 in human gallbladder carcinoma inhibits tumor growth *in vitro* and *in vivo*. *Sci. Rep*. **6**, 28833; doi: 10.1038/srep28833 (2016).

## Supplementary Material

Supplementary Information
