# Supplementary material for: Downregulation of stathmin 1 in human gallbladder carcinoma inhibits tumor growth in vitro and in vivo
Source: Sci Rep. 2016 Jun 28;6:28833. doi: 10.1038/srep28833 (PMC4923895; doi:10.1038/srep28833)
Supplement: Supplementary Information [file srep28833-s1.doc]

**Title: Downregulation of stathmin 1 in human gallbladder carcinoma inhibits tumor growth *in vitro* and *in vivo***

Author list: Jiwen Wang^1,2+^, Yanli Yao^2+^, Yue Ming^3+^, Sheng Shen^1^, Nan Wu^4,5^, Jiaqi Liu^6^, Han Liu^1^, Tao Suo^1^, Hongtao Pan^1^, Dexiang Zhang^7^, Kan Ding^2*^, Houbao Liu^1*^

**Table S1. The clinical characteristics of 37 GBC cases**

| Patient No. | Age | Gender | Pathologic Grade | Tumor size | T stage | N stage | M stage | Pathologic Stage | Pathological type |
| --- | --- | --- | --- | --- | --- | --- | --- | --- | --- |
| 1# | 70 | Female | Ⅱ | 8×7×6 | T3 | N0 | M1 | 4B | Adenocarcinoma |
| 2# | 83 | Female | Ⅱ | 2.5×1.5×0.6 | T2 | N0 | M0 | 2 | Adenocarcinoma |
| 3# | 78 | Female | Ⅱ | 7×5×4 | T2 | N0 | M0 | 2 | Adenocarcinoma |
| 4# | 60 | Female | Ⅱ | 6.53×1.5 | T1 | N0 | M0 | 1 | Adenocarcinoma |
| 5# | 45 | Female | Ⅱ | 3×3×2 | T2 | N0 | M0 | 2 | Adenocarcinoma |
| 6# | 79 | Female | Ⅱ | 6×4×4 | T2 | Nx | M0 | 2-4 | Adenocarcinoma |
| 7# | 77 | Female | Ⅱ-Ⅲ | 6.5×3.5×3 | T2 | N0 | M0 | 2 | Adenocarcinoma |
| 8# | 65 | Female | Ⅱ-Ⅲ | 2×1.5×1.5 | T2 | N1 | M0 | 3 | Adenocarcinoma |
| 9# | 57 | Female | Ⅲ | 8×5×4 | T3 | N0 | M0 | 3A | Adenocarcinoma |
| 10# | 72 | Female | Ⅱ | 7×5×5 | T3 | Nx | M0 | 3-4 | Adenosquamous carcinoma |
| 11# | 56 | Female | Ⅱ-Ⅲ | 4×3.5×1 | T2 | N0 | M0 | 2 | Adenosquamous carcinoma |
| 12# | 50 | Male | Ⅱ-Ⅲ | 5.3×4×2 | T3 | Nx | M0 | 3-4 | Adenosquamous carcinoma |
| 13 | 53 | Male | Ⅰ | 5×5×0.5 | T1 | N0 | M0 | 1 | Adenocarcinoma |
| 14 | 47 | Female | Ⅱ | 6×3.5×2 | T3 | N0 | M0 | 3A | Adenocarcinoma |
| 15 | 70 | Female | Ⅱ | 5×5×1 | T2 | N0 | M0 | 2 | Adenocarcinoma |
| 16 | 53 | Male | Ⅱ | 7×4×1 | T2 | N0 | M0 | 2 | Adenocarcinoma |
| 17 | 75 | Female | Ⅱ-Ⅲ | 5×3×1 | T3 | N0 | M1 | 4B | Adenocarcinoma |
| 18 | 74 | Male | Ⅱ-Ⅲ | 3.5×3×2 | T3 | N0 | M0 | 3A | Adenocarcinoma |
| 19 | 77 | Female | Ⅱ | 3.5×3×3 | T3 | N0 | M0 | 3A | Adenocarcinoma |
| 20 | 57 | Female | Ⅱ-Ⅲ | 6×4×1.5 | T2 | N1-2 | M1 | 4B | Adenocarcinoma |
| 21 | 54 | Female | Ⅱ | 6×6×4 | T3 | N0 | M0 | 3A | Adenocarcinoma |
| 22 | 73 | Male | Ⅱ | 4×3×3 | T3 | N0 | M0 | 3A | Adenocarcinoma |
| 23 | 64 | Female | Ⅱ | 5×4.5×4 | T3 | N1-2 | M0 | 3B－4B | Adenocarcinoma |
| 24 | 59 | Male | Ⅱ | 5×3×2 | T2 | N1 | M0 | 3B | Adenocarcinoma |
| 25 | 77 | Male | Ⅱ-Ⅲ | 3×2.5×1.5 | T3 | N0 | M0 | 3A | Adenocarcinoma |
| 26 | 51 | Male | Ⅱ-Ⅲ | 4.5×4×2.5 | T2 | N0 | M0 | 2 | Adenocarcinoma |
| 27 | 75 | Male | Ⅱ-Ⅲ | 5×4×4 | T3 | Nx | M0 | 3-4 | Adenocarcinoma |
| 28 | 67 | Male | Ⅱ-Ⅲ | 5×4×1.5 | T3 | N0 | M0 | 3A | Adenocarcinoma |
| 29 | 74 | Male | Ⅱ-Ⅲ | 6.5×5×5 | T3 | N1 | M0 | 3B | Adenocarcinoma |
| 30 | 60 | Female | Ⅲ | 4×3×3 | T3 | N0 | M0 | 3A | Adenocarcinoma |
| 31 | 60 | Male | Ⅲ | 3×2.5×1 | T3 | N0 | M1 | 4B | Adenocarcinoma |
| 32 | 76 | Female | Ⅲ | 4×3.5×3 | T2 | N0 | M0 | 2 | Adenocarcinoma |
| 33 | 69 | Female | Ⅲ | 6.5*6*5.5cm | T3 | Nx | M1 | 4B | Adenocarcinoma |
| 34 | 82 | Female | Ⅲ | 6*5*4cm | T2 | N1 | M0 | 3B | Adenocarcinoma |
| 35 | 56 | Female | Ⅱ-Ⅲ | 7.5*5*3.5cm | T4 | N0 | M0 | 4A | Adenosquamous carcinoma |
| 36 | 65 | Female | Ⅲ | 5*4*3.5cm | T2 | N0 | M0 | 2 | Adenosquamous carcinoma |
| 37 | 63 | Female | Ⅰ | 7*6*5 | T3 | N0 | M0 | 3A | Adenosquamous carcinoma |

#: The 12 specimens were the samples used in the first study, as shown in Fig. 1a-g. The other 25 specimens were evaluated in the additional experiment.

Table S2. The relationship between STMN1 expression and pathological grading/TNM stage

|  | STMN1 low expression^a^ | STMN1 high expression^a^ | *P*-value^b^ |
| --- | --- | --- | --- |
|  | (n = 14) | (n = 23) |  |
| Pathological Grading |  |  | 0.018 |
| Ⅰ,Ⅱ | 10 | 7 |  |
| Ⅱ~Ⅲ, Ⅲ | 4 | 16 |  |
| TNM Stage |  |  | 0.338 |
| 1-2 | 6 | 7 |  |
| 3-4 | 8 | 16 |  |

a: STMN1 high expression was defined by score index＞4, STMN1 low expression was defined by score index ≤ 4;

b: χ-square test

**Table S3.** **The primers for qRT-PCR.**

| No. | Gene | Forward primer | Reverse primer |
| --- | --- | --- | --- |
| 1 | STMN1 | TCGCTACGTTCAAGCTGTGT | ACGTCGAACTTGACCCATCC |
| 2 | GAPDH | CTCTCTGCTCCTCCTGTTCGAC | TGAGCGATGTGGCTCGGCT |

STMN1 expression levels of the two cell lines were similar according to the results of Western blot (Figure 1h) and qPCR (Supplementary Table S4). The results of growth, migration and invasion assays showed that the aggressiveness of these two cell lines was decreased significantly after the silence of STMN1. The aggressiveness variations of GBC-SD caused by shSTMN1-1 were significantly more than that of SGC-996 (Supplementary Table S4).

There are many gene mutations and expression levels influencing the aggressiveness ability of cells. STMN1 is just one of them. Besides, the gene mutation and expression spectrums were different among different cells. Thus, the role of the same gene is not exactly the same in different cells. The expression level of STMN1 plays a more important role in SGC-996 than in GBC-SD. This may well be a phenomenon worthy of study in the future. On the basis of an increase in cell types, we can research the mechanism more clearly.

**Table S4. The variations of STMN1 expression and aggressiveness of GBC cell lines.**

|  | Aggressiveness change (means±SD) | | |
| --- | --- | --- | --- |
| Cell lines | SGC-996 | GBC-SD | P-value |
| mRNA STMN1 | 13.42±4.63 | 19.87±5.09 | 0.1796 |
| Growth curve day 1 | 103.15±8.36 | 100.39±5.37 | 0.6556 |
| Growth curve day 2 | 89.10±0.28 | 91.36±7.22 | 0.6163 |
| Growth curve day 3 | 82.81±7.57 | 92.04±2.28 | 0.1134 |
| Growth curve day 4 | 72.23±3.54 | 80.10±3.02 | 0.0428 * |
| Growth curve day 5 | 61.47±2.56 | 74.39±2.47 | 0.0036 ** |
| Growth curve day 6 | 54.96±2.06 | 63.12±2.52 | 0.0122 * |
| Invasion | 27.66±1.56 | 70.46±2.44 | 0.00001 ** |
| Migration | 26.81±0.43 | 68.71±4.49 | 0.0001 ** |

Significance was determined by Student’s *t*-test. *: *p* ＜ 0.05; **: *p* ＜ 0.01.
